# Supplementary material for: Parallel Acquisition of Plasma Membrane Ultrastructure and Cytosolic Protein Localisation in Cultured Cells via Correlated Immunogold SEM
Source: Cells. 2020 May 26;9(6):1329. doi: 10.3390/cells9061329 (PMC7349049; doi:10.3390/cells9061329)
Supplement: Supplementary file 1 [file cells-09-01329-s001.pdf]

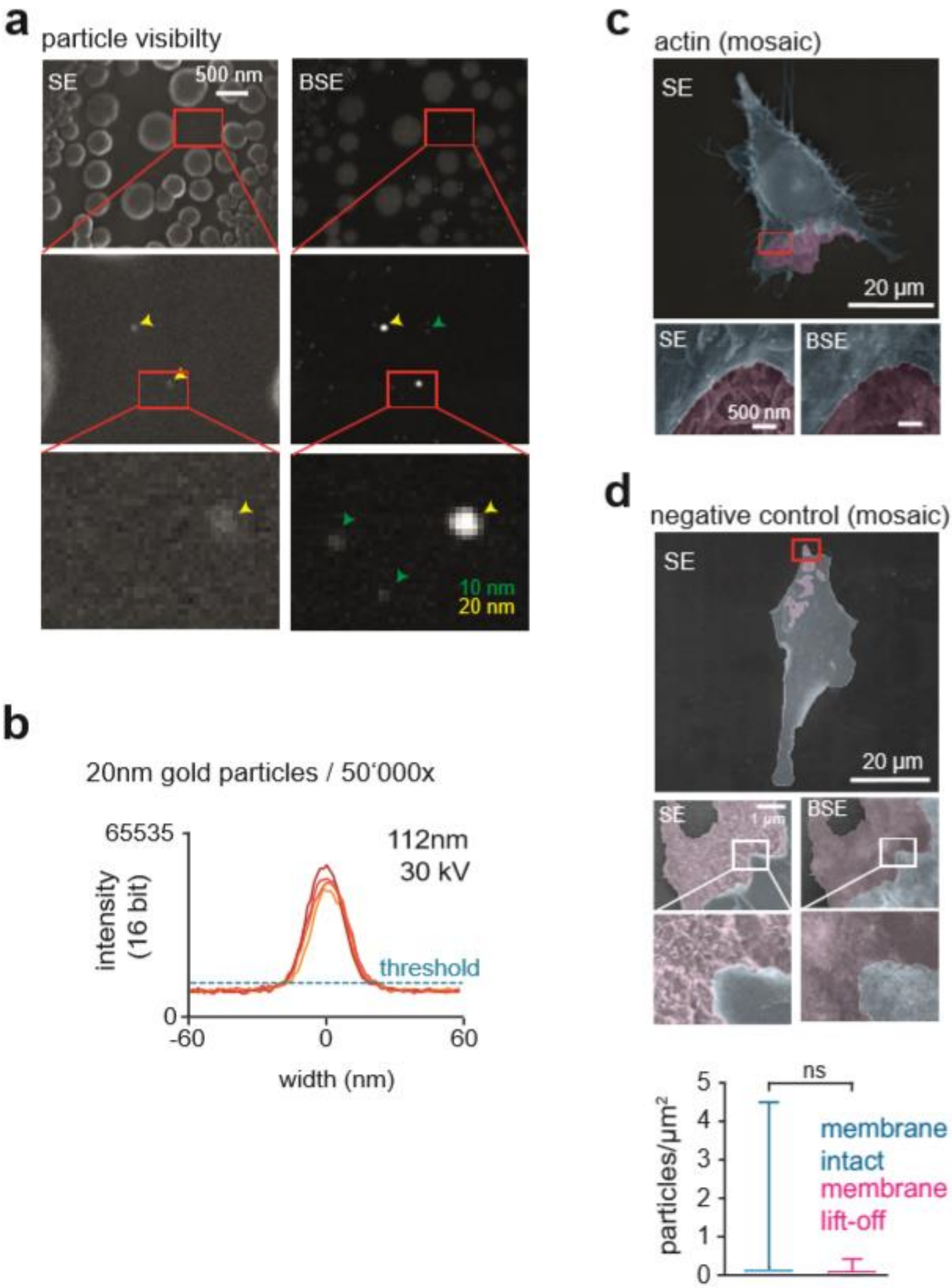

Figure S1. Validation of gold particle detection. (**a**) 10 nm and 20 nm gold particles can be separated by BSE. Latex balls were spread together with 10 nm (green) and 20 nm (yellow) gold-conjugated antibodies and imaged in SE (left) and BSE (right) mode. Images were acquired at 20,000× magnification (3.62 nm per pixel) with an acceleration voltage of 30 kV. Note, in zoom-in (bottom) that gold particles with a size of 10 nm (green arrows) and 20 nm (yellow arrows) can easily be distinguished. (**b**) Line scans of 20 nm gold particles that were coated with 112 nm carbon and imaged (16 bit resolution) at an acceleration voltage of 30 kV and 50,000× magnification. (**c**) Partially unroofed samples show no obscuring of gold particle signal directed against actin by membrane (pink: Unroofed; cyan: Intact membrane). All images were acquired with an acceleration voltage of 30 kV and magnification of 1,500× (top, 48.3 nm per pixel) and 25,000× (bottom, 2.89 nm per pixel). (**d**) No

masking or trapping of immunogold particle signal by membrane. Samples were prepared in the absence of a primary antibody (neg. control). Again, in some areas weakly adhesive tape was used to partially unroof (pink) the cell. Samples were imaged in SE (left) and BSE (right) mode. Images were acquired at 800× (top, 90.6 nm per pixel) and 20,000× magnification (bottom, 3.62 nm per pixel) with an acceleration voltage of 30 kV. For statistical analysis, gold particles were counted in intact membrane areas (cyan) and in unroofed areas (pink) (negative intact, n = 17 images; negative lift-off, n = 15 images; t-test (WC) $p < 0.2479$; MW $p = 0.0716$). Scale bar: (a) 500 nm, (c) 20 μm, 500 nm, (d) 20 μm, 1 μm.

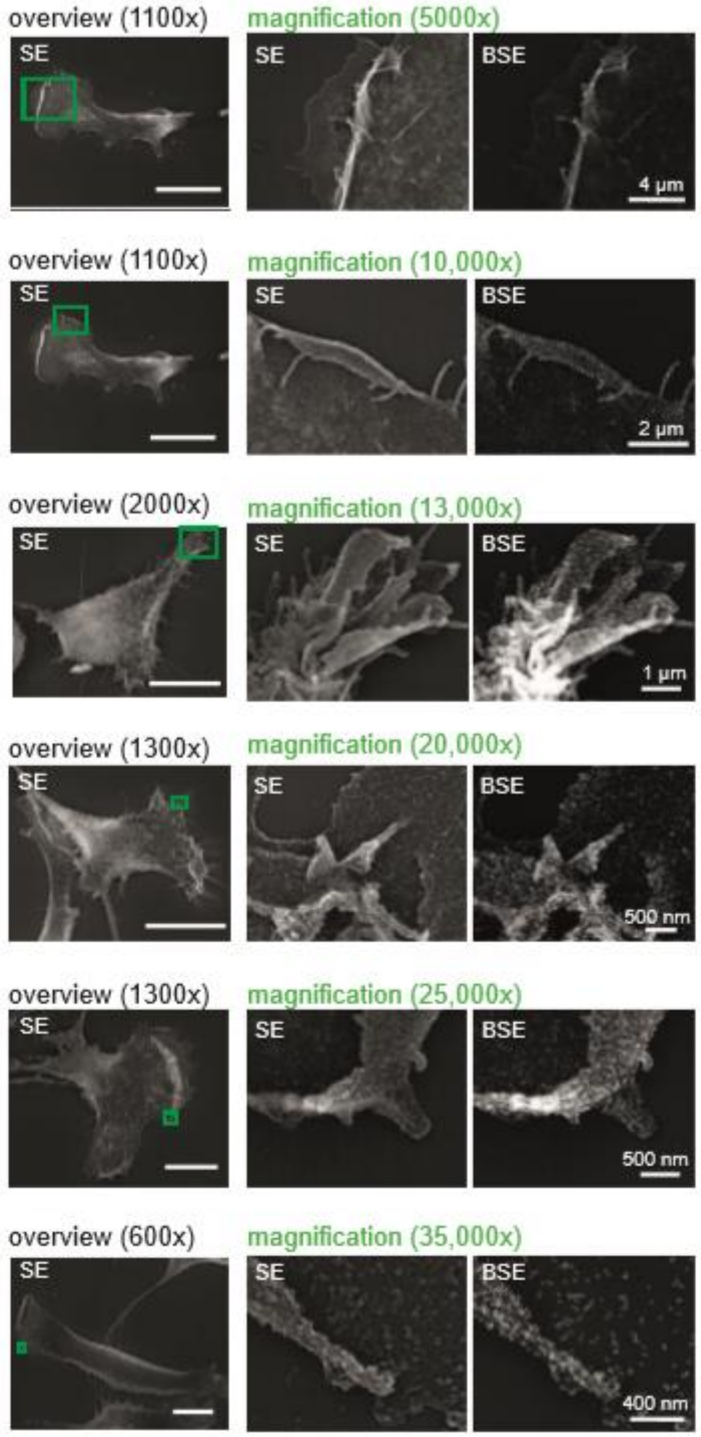

Figure S2. Representative examples of curvature-sensitive I-BAR domain localisation in fixed samples. Samples were transfected with a biosensor composed of the I-BAR domain tagged to GFP, labelled with a primary antibody against GFP and a secondary antibody conjugated with 20 nm gold particles. For each image, an overview (left, black) and zoom-in of the highlighted region (right, green) are shown. To the right, examples of I-BAR localisation imaged in SE and BSE mode at an acceleration voltage of 30 kV and (from top to bottom) a magnification of 5,000× (14,49 nm per pixel), 10,000× (7.25 nm per pixel), 13,000× (5,57 nm per pixel), 20,000× (3,62 nm per pixel), 25,000× (2,90 nm per pixel) and 35,000× (2,07 nm per pixel). Scale bars: 15 µm, 4 µm, 2 µm, 1 µm, 500 nm, 400 nm.
